# Trends in shaken baby syndrome diagnosis codes among young children hospitalized for abuse

**DOI:** 10.1186/s40621-021-00334-w

**Published:** 2021-07-19

**Authors:** Aislinn Conrad, Brandon Butcher, Resmiye Oral, Megan Ronnenberg, Corinne Peek-Asa

**Affiliations:** 1 University of Iowa School of Social Work, 308 North Hall, Iowa City, IA 52246 USA; 2 Injury Prevention Research Center and Department of Biostatistics, University of Iowa College of Public Health, Iowa City, IA USA; 3 Geisel School of Medicine, Children’s Hospital at Dartmouth, Lebanon, USA; 4 Injury Prevention Research Center and Occupational and Environmental Health, University of Iowa College of Public Health, Iowa City, IA USA

**Keywords:** Shaken baby syndrome, Abusive head trauma, Child abuse, Trends, Secondary data

## Abstract

**Objective:**

To investigate national trends of SBS diagnosis codes and how trends varied among patient and hospital characteristics.

**Methods:**

We examined possible SBS, confirmed SBS, and non-SBS abuse diagnosis codes among children age three and younger who were hospitalized for abuse between 1998 and 2014 using a secondary analysis of the National Inpatient Sample, the largest US all-payer inpatient care database (*N* = 66,854). A baseline category logit model was used based on a quasi-likelihood approach (QIC) with an independent working correlation structure.

**Results:**

The rate (per 100,000 census population of children age 3 and younger) of confirmed and possible SBS diagnosis codes was 5.4 (± 0.3) between 1998 and 2014, whereas the rate of non-SBS abuse was 19.6 (± 1.0). The rate of confirmed SBS diagnosis codes increased from 3.8 (± 0.3) in 1998 to 5.1 (± 0.9) in 2005, and decreased to 1.3 (± 0.2) in 2014. Possible SBS diagnosis codes were 0.6 (± 0.2) in 1998, increasing to 2.4 (± 0.4) in 2014. Confirmed SBS diagnosis codes have declined since 2002, while possible SBS diagnosis codes have increased. All abuse types were more frequent among infants, males, children from low-income homes, and urban teaching hospitals.

**Conclusions:**

We investigated seventeen-year trends of SBS diagnosis codes among young children hospitalized for abuse. The discrepancy between trends in possible and confirmed SBS diagnosis codes suggests differences in norms for utilizing SBS diagnosis codes, which has implications for which hospital admissions are coded as AHT. Future research should investigate processes for using SBS diagnosis codes and whether all codes associated with abusive head injuries in young children are classified as AHT. Our findings also highlight the relativity defining and applying SBS diagnosis codes to children admitted to the hospital for shaking injuries. Medical professionals find utility in using SBS diagnosis codes, though may be more apt to apply codes related to possible SBS diagnosis codes in children presenting with abusive head injuries. Clarifying norms for SBS diagnosis codes and refining definitions for AHT diagnosis will ensure that young children presenting with, and coded for, abusive head injuries are included in overall counts of AHT based on secondary data of diagnosis codes. This baseline data, an essential component of child abuse surveillance, will enable ongoing efforts to track, prevent, and reduce child abuse.

**Supplementary Information:**

The online version contains supplementary material available at 10.1186/s40621-021-00334-w.

## Introduction

Every year, approximately 33 per 100,000 infants are diagnosed with abusive head trauma in US hospitals (Shanahan et al. 2013), leading to mild to moderate behavioral and cognitive problems among abuse survivors, and lifelong disability or death in severe instances (Duhaime and Christian 2019). Pediatric abusive head trauma (AHT) includes injuries to children’s skulls or intracranial contents that occur when perpetrators violently shake young children, with or without intentional impact (Lopes et al. 2013). The incidence of AHT is likely underestimated due to our ability to surveil AHT and the secrecy, stigma, and shame associated with child abuse (Fallon et al. 2010; Sedlak et al. 2010). Mild cases of AHT may go unrecognized while moderate to severe cases require hospitalization where surveillance occurs, often using 15 diagnosis codes recommended by the Centers for Disease Control (CDC) (Center for Disease Control and Prevention 2012). Though the annual incidence of AHT likely exceeds the use of AHT diagnosis codes within hospitals, inpatient data are ideal for public health surveillance; hospital datasets provide consistent and reliable sampling frames (i.e., all children hospitalized for AHT), and uniform measures of AHT (i.e., diagnosis codes from International Classification of Disease, Clinical Modification [ICD-CM]) (Wirtz and Trent 2008).

AHT is the official term for intentional abusive head injuries in children, yet researchers and medical professionals formerly ascribed these injuries to *shaken baby syndrome* (SBS). Whereas SBS indicates one specific mechanism of injury, i.e., shaking, AHT includes a broader range of injury mechanisms, leading the American Academy of Pediatrics (AAP) and CDC’s decision to adopt AHT terminology instead of SBS (Center for Disease Control and Prevention 2012; Christian and Block 2010). Although there is no consensus among researchers that shaking alone causes AHT-like injuries, about 35% of AHT hospitalizations include a diagnosis code for SBS (Parks et al. 2012). The continued reliance on SBS codes suggests that medical professionals find utility in the diagnosis, yet little is known about the use of SBS diagnosis codes per year or over time.

These knowledge gaps are problematic for two reasons. First, researchers do not know whether trends in SBS diagnosis codes correspond with the stable trends in AHT diagnosis codes observed 2003–2008 among children (< 5 years old) (Hymel et al. 1998) and 2000–2009 among infants (< 1 year old) (Shanahan et al. 2013). Evidence that SBS diagnosis codes are declining over time would suggest that SBS diagnosis codes are decreasing as well, which offers AAP and CDC confidence about the effectiveness and accuracy of their messaging about AHT. Second, researchers do not know the extent to which SBS diagnosis codes are used every year, nor the hospital and patient characteristics associated with this diagnosis code. Such evidence would highlight inconsistencies between current and desired use of diagnosis codes for SBS and help policymakers align current coding practices with guidelines for coding SBS. In response, we examined trends in SBS diagnosis codes (ICD-9 code 995.55) between 1998 and 2014.

Another issue related to SBS diagnosis codes pertains to accuracy. Medical professionals who diagnose SBS rely largely on a unique pattern of injuries they attribute to shaking, including subdural hematoma, retinal hemorrhages and encephalopathy, otherwise known as the so-called “triad” (Squier 2008, 2011). However, researchers do not know whether the SBS diagnostic code (995.55) captures all abusive head injuries related to the “triad.” In absence of this information, some pediatric abusive head injuries may remain unclassified as either SBS or AHT. The CDC definitions for both probable and definite AHT, for example, exclude retinal hemorrhages and convulsions without a seizure disorder, two symptoms typically associated with SBS (Hymel et al. 1998). This is problematic considering that up to 40% of AHT hospitalizations with an SBS diagnosis would be considered non-AHT abuse without the SBS diagnostic code (Hymel et al. 1998). In response, we used diagnostic codes from ICD-9 to develop measures of possible and confirmed SBS. For possible SBS, we included ICD-9 codes for retinal hemorrhages and convulsions without a seizure disorder, whereas our measure of confirmed SBS included only the ICD-9 code for shaken baby syndrome (995.55) among young children hospitalized for child abuse.

In all, few, if any, researchers have examined the prevalence of SBS diagnosis codes or how the use of codes are trending over time. In the absence of this data, researchers will not know the extent to which SBS is diagnosed, or the patient and hospital characteristics associated with SBS diagnosis. Therefore, the purpose of our study was to investigate trends in SBS diagnosis codes among a nationally representative sample of children age three and younger who were hospitalized for abuse. Using 1998–2014 data from the National Inpatient Sample (NIS) (Agency for Healthcare Research and Quality (AHRC) 2012), we described the demographic characteristics of children hospitalized with possible and confirmed SBS diagnosis codes, calculated the annual rate of non-SBS abuse and possible and confirmed SBS diagnosis codes, and investigated how trends varied among patient and hospital characteristics.

Through our findings on trends in SBS diagnosis, we can determine whether the use of the SBS code (995.55) has declined since the AAP’s recommendation in 2009, and whether the SBS code accounts for all possible SBS diagnosis codes, which are not currently captured within the 15 AHT diagnosis codes.

## Method

### Sample

Data from 1998 to 2014 from the National Inpatient Sample (NIS) (Lau et al. 2005) were used for this retrospective study. The NIS is the largest all-payer, inpatient care database in the US and is maintained by the Healthcare Utilization project. We identified our sample of children 3 years of age or younger who were hospitalized for abuse using the ICD-9 diagnosis and external cause of injury codes in the Additional file 1. These codes describe the type of injury and perpetrator. We examined SBS diagnosis codes among young children hospitalized for any form of abuse for two reasons. First, though associations between physical abuse (e.g. SBS) and non-physical forms of abuse may seem surprising, physical and non-physical abuse co-occurs among 30 to 90% of abused children (Claussen and Crittenden 1991; Kim et al. 2017a; McGee et al. 1995; Lau et al. 2005; Boxer and Terranova 2008). Second, healthcare providers may misdiagnose or fail to diagnose earlier instances of child physical abuse preceding severe incidences of abuse (Christian 2015; King et al. 2006). These findings suggest that young children with SBS diagnosis codes may also present to the hospital with a non-physical form of abuse.

Although data from 2015 and 2016 are available, we did not use these data due to the switch from ICD-9-CM to ICD-10-CM diagnosis standards in 2015. This change led to substantive shifts in abuse coding, meaning that changes in abuse frequency could not be discerned from changes in the coding standard. All analyses incorporated sampling weights to provide nationally representative estimates.

### Measures

Our analysis included four measures of child abuse within our sample: Non-SBS abuse, confirmed SBS, possible SBS, and total SBS. We defined these three categories as follows:

- *Confirmed SBS Abuse Diagnosis:* The presence of diagnosis code 995.55.
- *Possible SBS Abuse:* The absence of diagnosis code 995.55 and the presence of physical abuse (995.54), Type 1 internal traumatic brain injury (TBI; 800,801,803,804(.1–.4,.6–.9,.03–.05,.53–.55), 850(.2–.4), 851–854, 950(.1–.3)), retinal hemorrhage (362.81), and convulsions not associated with a seizure disorder (780.39) (see Additional file 1 for explanation of codes).
- *Non-SBS Abuse*: Remaining cases that did not meet the criteria of Confirmed or Possible SBS.
- *Total SBS Abuse*: The sum of confirmed and possible incidences of SBS.

Per the Barell injury matrix (Bergen et al. 2008), Type 1 TBI diagnosis codes include primary intracranial injury, moderate to prolonged loss of consciousness, shaken infant syndrome, or injuries to the optic nerve pathways. We included retinal hemorrhage and convulsions not related to a seizure disorder because they indicate acceleration/deceleration of the head (McGee et al. 1995), yet are not included in the CDC’s recommended codes for AHT (Center for Disease Control and Prevention 2012). While traumatic brain injury from impact is not precluded, this combination of codes indicates that children were subjected to high-force acceleration injury, characteristic of SBS.

### Analytic strategy

We calculated the annual prevalence of SBS hospital diagnosis codes from 1998 through 2014 for confirmed SBS, possible SBS, total SBS, and non-SBS abuse. Our primary objective was to estimate trends in the probability of confirmed and possible SBS diagnosis codes among the population of children age 3 and younger who were hospitalized for abuse. A Chi-square test was used to test for independence of possible and confirmed SBS diagnosis codes from patient and hospital characteristics. Differences between total SBS and all other abuse diagnosis codes (non-SBS abuse) were also estimated for the purpose of comparing possible and confirmed SBS diagnosis codes to non-SBS diagnosis codes (Table 1).

**Table 1:** National hospital admissions for children up to three years, 1998–2014 (*N* = 66,854), patient- and hospital-level descriptive statistics, by type of abuse

Variable	Possible SBS N (%)	Confirmed SBS N (%)	Chi-square	Total SBS N (%)	Non-SBS Abuse N (%)	Chi-square
	4209 (29.4)	10,083 (70.6)	*P* value	14,292 (21.4)	52,562 (79.2)	*P* value
*Rates (per 100,00 population)*						
Total rate of abuse	1.6 (± 0.1)	3.8 (± 0.3)	–	4.5 (± 0.3)	19.6 (± 1.0)	–
Rate of abuse per year						
1998	0.6 (± 0.2)	3.6 (± 0.6)	–	4.1 (± 0.7)	17.8 (± 2.5)	–
1999	1.1 (± 0.3)	6.4 (± 1.0)	–	7.5 (± 1.2)	20.1 (± 2.3)	–
2000	0.6 (± 0.2)	4.6 (± 0.8)	–	5.2 (± 0.9)	16.6 (± 2.0)	–
2001	1.0 (± 0.3)	4.5 (± 0.8)	–	5.5 (± 1.1)	19.1 (± 3.1)	–
2002	0.7 (± 0.2)	4.3 (± 0.8)	–	5.0 (± 0.9)	17.5 (± 2.3)	–
2003	1.1 (± 0.3)	5.0 (± 0.8)	–	6.1 (± 1.0)	18.2 (± 2.6)	–
2004	1.3 (± 0.4)	4.8 (± 0.9)	–	6.1 (± 1.2)	18.1 (± 2.7)	–
2005	2.0 (± 0.6)	5.1 (± 0.9)	–	7.1 (± 1.3)	22.7 (± 3.6)	–
2006	1.3 (± 0.4)	4.5 (± 0.8)	–	5.9 (± 1.0)	17.9 (± 2.5)	–
2007	2.0 (± 0.6)	3.8 (± 0.7)	–	5.8 (± 1.1)	18.7 (± 3.5)	–
2008	1.2 (± 0.4)	3.8 (± 0.6)	–	5.0 (± 0.9)	17.3 (± 2.9)	–
2009	1.9 (± 0.5)	3.1 (± 0.6)	–	5.0 (± 1.0)	17.3 (± 2.8)	–
2010	1.9 (± 0.5)	3.6 (± 0.6)	–	5.6 (± 0.9)	25.8 (± 4.2)	–
2011	2.6 (± 0.7)	1.5 (± 0.3)	–	4.1 (± 0.9)	18.8 (± 3.8)	–
2012	2.3 (± 0.4)	2.2 (± 0.3)	–	4.4 (± 0.5)	21.4 (± 2.0)	–
2013	2.5 (± 0.4)	2.0 (± 0.3)	–	4.5 (± 0.5)	23.0 (± 2.0)	–
2014	2.4 (± 0.4)	1.3 (± 0.2)	–	3.7 (± 0.4)	22.5 (± 2.0)	–
*Patient characteristics*						
Age						
< 1	2744 (65.2)	8162 (80.9)	< 0.001	10,906 (76.3)	33,428 (63.6)	< 0.001
1	700 (16.6)	1160 (11.5)		1860 (13)	8705 (16.6)	
2/3	765 (18.2)	761 (7.5)		1526 (10.7)	10,429 (19.8)	
Hospital mortality						
Yes	571 (13.6)	1271 (8.9)	0.467	1842 (12.9)	1597 (3.0)	< 0.001
No	3637 (86.4)	8812 (61.7)		12,450 (87.1)	50,964 (97.0)	
Sex						
Female	1530 (36.4)	4067 (40.3)	0.074	5597 (39.2)	22,486 (42.8)	0.007
Male	2679 (63.6)	6016 (59.7)		8695 (60.8)	30,075 (57.2)	
Race						
Asian or Pacific Islander	63 (1.5)	260 (2.6)	0.240	323 (2.3)	467 (0.9)	< 0.001
Black	853 (20.3)	1992 (19.8)		2845 (19.9)	12,774 (24.3)	
Hispanic	718 (17.1)	1614 (16)		2332 (16.3)	9261 (17.6)	
Native American	58 (1.4)	84 (0.8)		142 (1)	500 (1)	
Other	310 (7.4)	574 (5.7)		884 (6.2)	2632 (5)	
White	2206 (52.4)	5559 (55.1)		7765 (54.3)	26,928 (51.2)	
Income						
1st Quartile	1262 (30)	2558 (25.4)	0.001	3820 (26.7)	18,216 (34.7)	< 0.001
2nd Quartile	1349 (32.1)	2906 (28.8)		4255 (29.8)	15,490 (29.5)	
3rd Quartile	1037 (24.6)	2572 (25.5)		3609 (25.3)	11,703 (22.3)	
4th Quartile	560 (13.3)	2047 (20.3)		2607 (18.2)	7153 (13.6)	
*Hospital characteristics*						
Hospital type (since 2008)						
Government, nonfederal	331 (14.4)	413 (14.9)	0.067	744 (15.9)	3641 (14.6)	0.643
Private, investor-owned	98 (4.3)	243 (8.7)		341 (6.7)	1541 (6.7)	
Private, not-for-profit	1874 (81.4)	2123 (76.4)		3997 (77.4)	17,700 (78.6)	
Hospital size (bed count)						
Large	2380 (57.4)	6704 (66.7)	0.008	9084 (64.0)	32,545 (62.5)	0.479
Medium	1111 (26.8)	1944 (19.3)		3055 (21.5)	12,063 (23.2)	
Small	656 (15.8)	1398 (13.9)		2054 (14.5)	7455 (14.3)	
Hospital location / teaching						
Rural	45 (1.1)	328 (3.3)	0.005	373 (2.6)	3294 (6.3)	< 0.001
Urban non-teaching	263 (6.2)	1258 (12.5)		1521 (10.6)	6808 (13)	
Urban teaching	3901 (92.7)	8497 (84.3)		12,398 (86.7)	42,460 (80.8)	
Hospital region						
Midwest	1210 (28.7)	2707 (26.8)	0.128	3917 (27.4)	13,928 (26.5)	0.204
Northeast	436 (10.4)	1518 (15.1)		1954 (13.7)	8459 (16.1)	
South	1675 (39.8)	4177 (41.4)		5852 (40.9)	20,144 (38.3)	
West	888 (21.1)	1681 (16.7)		2569 (18)	10,029 (19.1)	

Trends were estimated overall and within categories of age and hospital size. Hospital size is categorized as large, medium, and small, although the NIS does not provide detailed information on how these categories are constructed. A baseline category logit model (Agresti 2013) was used based on a quasi-likelihood approach (QIC) with an independent working correlation structure in which non-SBS abuse is the reference category. The primary sampling unit in the NIS is the hospital; there are repeated observations within each hospital and across time. To account for the misspecification of the correlation structure, robust standard errors were used (i.e., “sandwich” estimators). Since there is a lack of research on SBS time trends, QIC was used to determine the form of the time trend for possible, confirmed, and non-SBS abuse: linear, quadratic, cubic, or treating time as categorical. A reduction in QIC of two-units was chosen a priori to indicate model preference. All statistical analyses were performed using SAS version 9.4.

## Results

### Sample characteristics

From 1998 to 2014, there were an estimated 66,854 total hospital admissions for child abuse among children 3 years of age and younger, with 52,562 hospital admissions for non-SBS abuse and 14,292 for SBS abuse, of which 10,083 were confirmed and 4209 were possible SBS. Total SBS admissions had greater distributions of young, male, White or higher income children, and hospital deaths than non-SBS admissions (*p* < .001; Table 1). Concerning age, 76.3% of total SBS admissions were under the age of one as compared to 63.6% of non-SBS admissions. In addition, moderate to higher income families (2-4th quartile) had greater representation among total SBS admissions compared to non-SBS abuse admissions (*p* <  0.001). The NIS categorizes patient income into quartiles based on the estimated median household income of residents in the patient’s ZIP code. Finally, whereas 3.0% of non-SBS admissions died in the hospital, 12.9% of total SBS admissions died.

In contrast, when comparing possible and confirmed SBS admissions, only distributions for age and income quartiles were statistically significant (*p* <  0.001). Although not statistically significant, 63.6% of possible SBS admissions were male compared to 59.7% of confirmed SBS admissions (*p* = 0.074), which is similar to total and non-SBS admissions. Lower income families (1st and 2nd income quartiles) had a higher representation among possible SBS admissions than confirmed SBS admissions (30% & 32.1% vs. 25.4% & 28.8%, respectively; *p* <  0.001).

There were statistically significant differences for one hospital characteristic for total SBS and non-SBS admissions: total SBS admissions were more frequent within urban teaching hospitals than non-SBS admissions (86.7% v. 80.8%, *p* <  0.001). Similarly, possible SBS was more frequent within urban teaching hospitals than confirmed SBS admissions (92.7% v. 84.3%, *p* <  0.01). Finally, confirmed SBS was most common in large hospitals, while possible SBS was more frequent in medium and small-sized hospital (*p* <  0.01).

### Time trends in rate of SBS

In Table 1, we summarize the rates (per 100,000 census population of children age 3 and younger (U.S. Census Bureau 2016a, b; U.S. Census Bureau 2018)) for non-SBS abuse, possible SBS, confirmed SBS, and total SBS. National yearly estimates of population-based rates of hospitalized child abuse or neglect were calculated using US Census Data with annual population estimates as the rate denominator. The overall rate of total SBS diagnosis codes was 5.4 (± 0.3) for every 100,000 children 3 years of age or younger from 1998 to 2014, whereas the rate of non-SBS abuse was 19.6 (± 1.0). The overall rate of confirmed SBS and possible SBS admissions was 3.8 (± 0.3) and 1.6 (± 0.1), respectively. The annual rate of non-SBS abuse admissions remained fairly stable, with the lowest rate of admissions in 2000 (16.6 ± 2.0) and the highest rate of admissions in 2010 (25.8 ± 4.2). However, the rate of admissions for confirmed SBS abuse increased from 3.6 (± 0.6) in 1998 to 5.1 (± 0.9) in 2005, at which point the rate steadily decreased to 1.3 (± 0.2) in 2014. Conversely, the rate of admissions for possible SBS increased gradually per year. In 1998, the rate of possible SBS admissions was 0.6 (± 0.2) and steadily increased to 2.4 (± 0.4) in 2014. Total SBS fluctuated but remained relatively stable, beginning at 4.1 (± 0.7) in 1998 and ending at 3.7 (± 0.4) in 2014.

#### Time trends in probability of type of abuse diagnosis

**Fig. 1:**
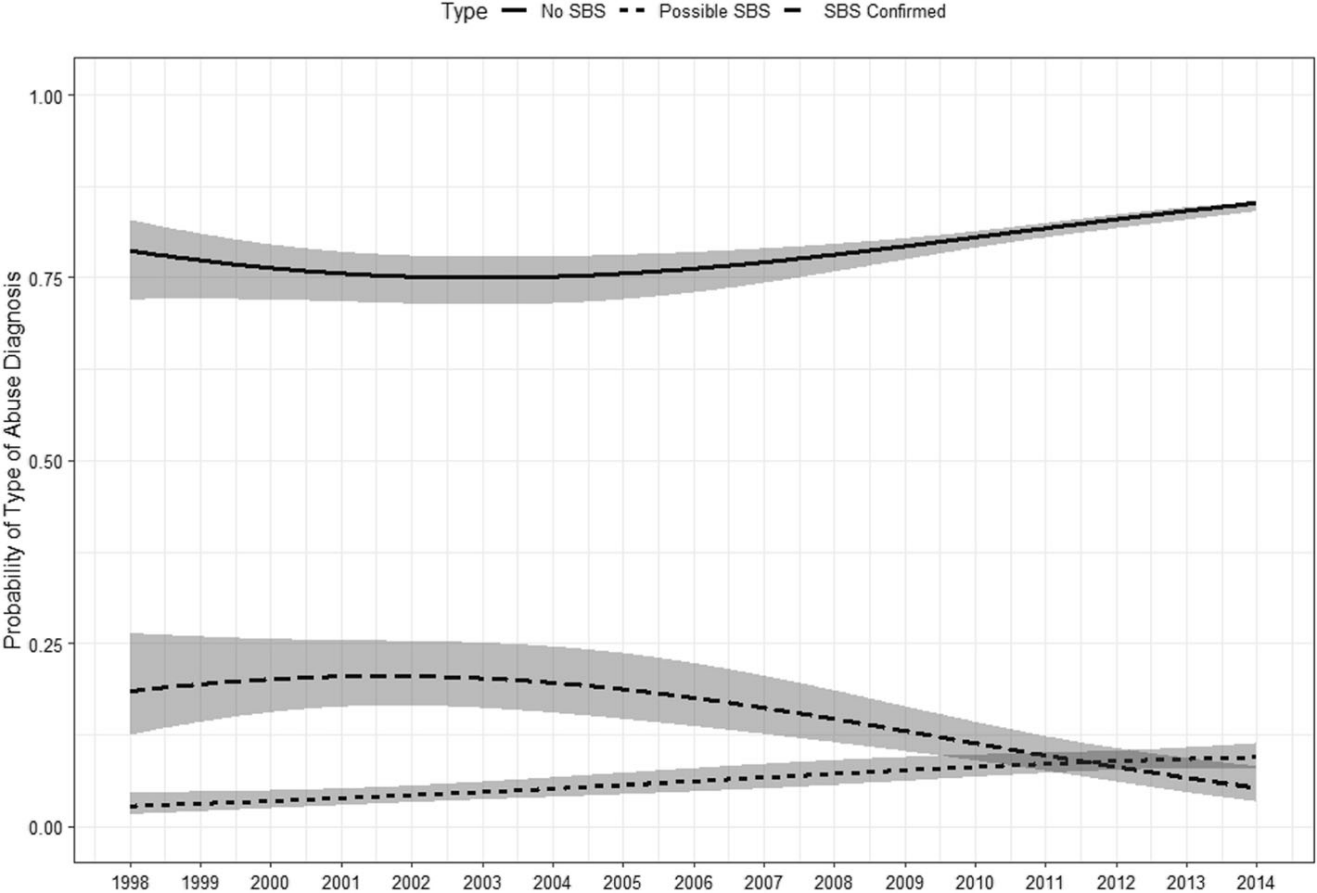
Overall time trend in the probability of diagnosing abuse as Confirmed SBS, Possible SBS, and Non-SBS abuse

**Fig. 2:**
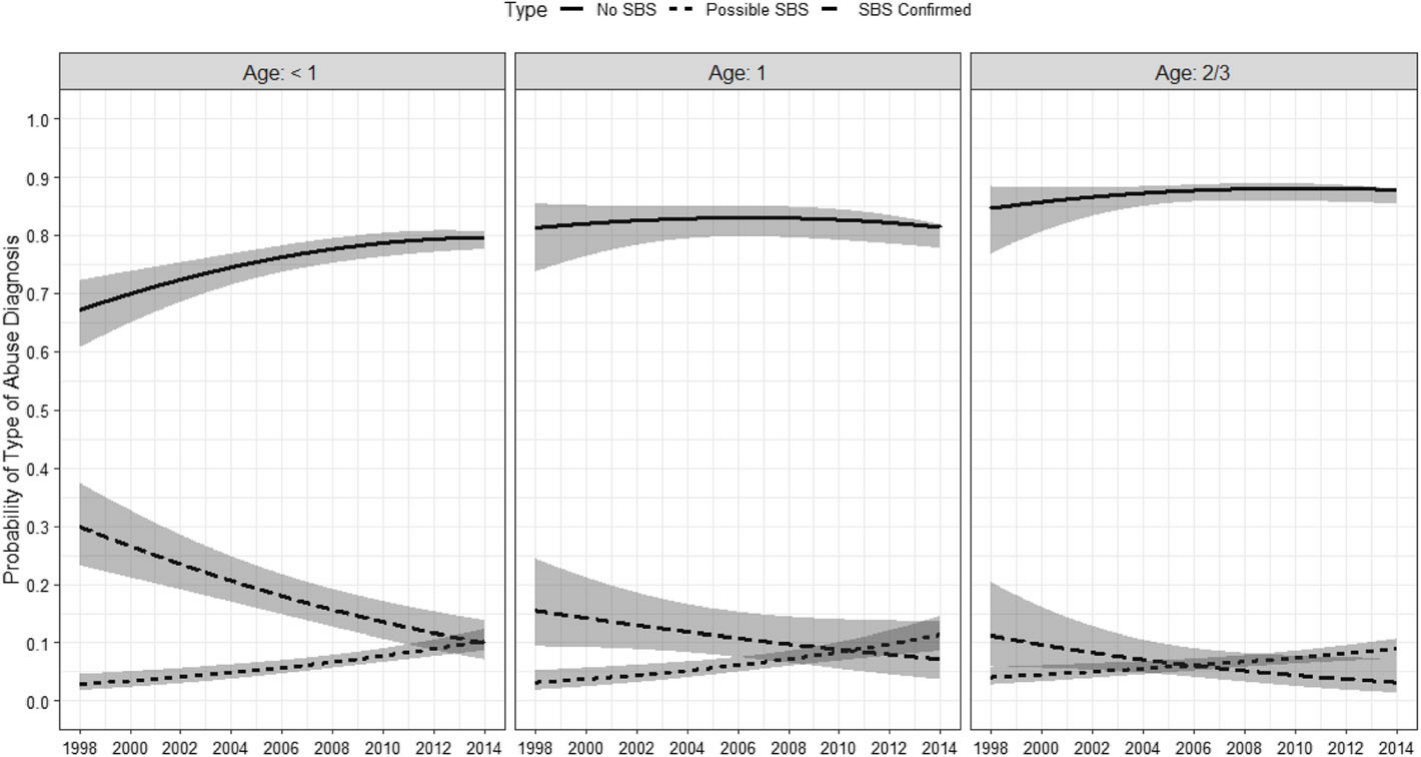
Overall time trend in the probability of diagnosing abuse as Confirmed SBS, Possible SBS, and Non-SBS abuse by age of patient

**Fig. 3:**
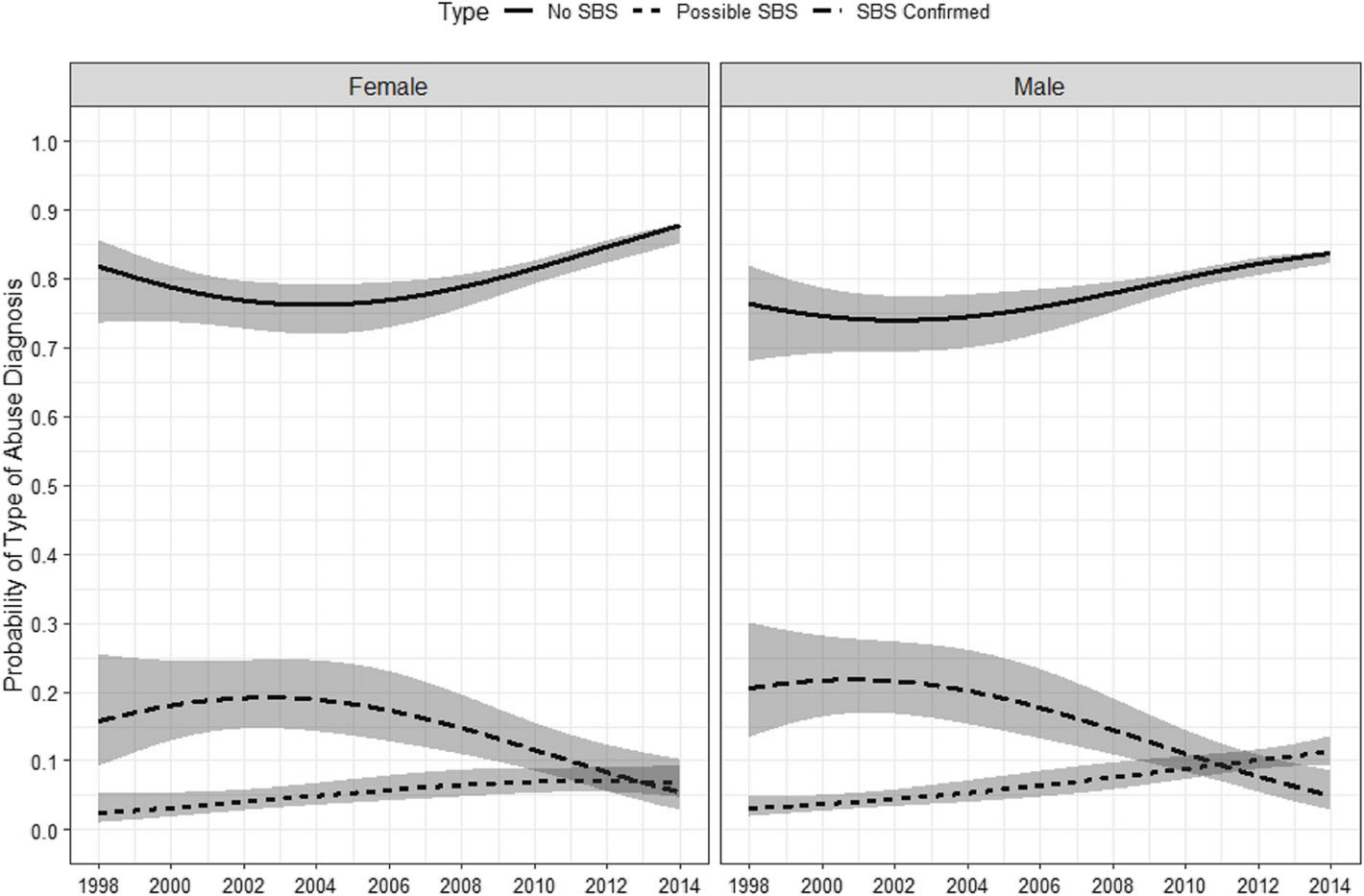
Overall time trend in the probability of diagnosing abuse as Confirmed SBS, Possible SBS, and Non-SBS abuse by sex

We conducted a baseline category logit model to examine whether the difference in trends for confirmed SBS, possible SBS, and the reference category, non-SBS abuse, were statistically significant. Figures 1, 2 and 3 indicate the results from these analyses (solid line and 95% confidence band). Non-SBS abuse includes all abuse cases not meeting criteria of confirmed or possible SBS. A statistically significant difference was found in the overall trends of the probability of possible SBS versus confirmed SBS (Fig. 1). Possible SBS gradually increased over time, whereas confirmed SBS slightly increased until 2001 and then decreased from 2002 to 2014 with the trends crossing over in 2011. In 1998, the estimated probability of possible SBS was 2.8% (95% CI: 1.7, 4.6), whereas the chance of confirmed SBS was 18.5% (95% CI: 12.5, 26.4). In 2014, the estimates for possible SBS and confirmed SBS were 9.4% (95% CI: 7.7, 11.3) and 5.3% (95% CI: 3.4, 8.2), respectively.

Figure 2 shows the statistically significant trends for possible SBS versus confirmed SBS by age group: < 1 year old, 1 year old, and 2 or 3 years old. The decrease in the probability of confirmed SBS was largest for infants (< 1 year old), declining from 29.9% (95% CI: 23.2, 37.4) in 1998 to 3.2% (95% CI: 1.4, 7.3%) in 2014, a decrease of 26.7%. Confirmed SBS diagnosis trends for infants (< 1 year old) overlapped with possible SBS in 2011. The decrease in confirmed SBS diagnosis trends was more attenuated for young toddlers (1 year old) than for older toddlers (2 or 3 years old) between 1998 and 2014, decreasing by 8.3% for young toddlers (15.5% [95%: 9.4, 24.4%] v. 7.2% [95% CI: 3.7, 13.6%], respectively), and decreasing by 4.0% for older toddlers (11.2% [95%: 5.9, 20.4%] v. 7.2% [95% CI: 3.2, 13.9%], respectively). The probability of possible SBS increased for all age groups in the same time period.

The trends for confirmed and possible SBS among females and males (Fig. 3) were similar to the overall trend (Fig. 1). For males, the probability of confirmed SBS increased from 20.5% (95% CI: 13.4, 30.1%) in 1998 to 21.8% (95% CI: 16.8, 27.7%) in 2000, and then decreased to 5.0% (95% CI: 2.9, 8.5%) in 2014. In contrast, the probability of a male receiving a possible SBS diagnosis increased gradually between 1998 to 2014 (3.1% [95% CI: 1.9, 4.8] v. 11.3% [95% CI: 9.3, 13.5], respectively). For females, the probability of a confirmed SBS diagnosis increased from 15.7% (95% CI: 9.2, 25.4%) in 1998 to 19.2% (95% CI: 14.7, 24.6%) in 2002, and then decreased to 5.6% (95% CI: 2.9, 10.2%) in 2014, whereas the probability of a female receiving a possible SBS diagnosis increased gradually by 4.3% from 1998 to 2014 (2.4% [95% CI: 1.1, 5.2%] v. 6.7% [95% CI: 4.8, 9.3%], respectively).

**Fig. 4:**
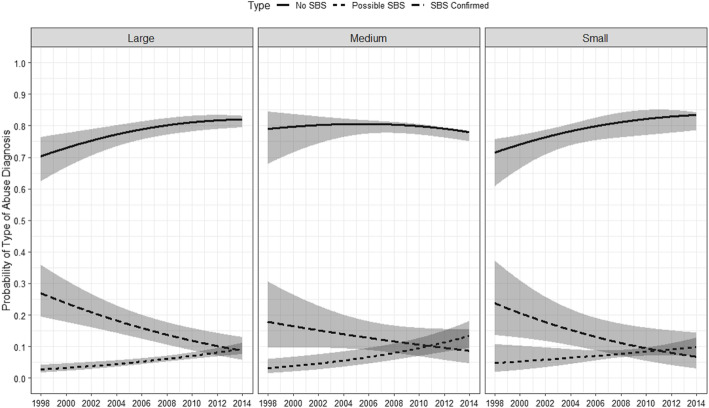
Overall time trend in the probability of diagnosing abuse as Confirmed SBS, Possible SBS, and Non-SBS abuse by hospital bed-size

Figure 4 shows the statistically significant trends for possible SBS versus confirmed SBS by hospital size (based on bed count). The most pronounced decrease in the probability of confirmed SBS was for large hospitals between 1998 and 2014 (26.9% [95% CI: 19.4, 35.9] v. 8.7% [95% CI: 5.7, 13.0%]). This decrease in confirmed SBS was less attenuated among medium-sized hospitals (17.8% [95%: 9.6, 30.6%] v. 8.7% [95% CI: 4.6, 15.4%]) than small hospitals (23.7% [95%: 13.6, 37.3%] v. 6.8% (95% CI: 3.0, 14.4%]) between 1998 and 2014. In contrast, the probability of possible SBS increased for each hospital size during the same time period.

## Discussion

AHT is a serious form of child physical abuse that happens when caregivers violently shake young children (Lopes et al. 2013). These abusive head injuries were classified as SBS until 2009, when the AAP and CDC recommended AHT diagnosis codes instead of SBS (Christian and Block 2010). Though non-fatal AHT diagnosis codes have stabilized or declined over time (Shanahan et al. 2013; Parks et al. 2012), little is known about SBS diagnosis trends. This knowledge gap is problematic considering that 40% of AHT hospitalizations with an SBS diagnosis would not be classified as AHT without an SBS diagnosis (Parks et al. 2012). Without knowing trends in SBS diagnosis codes, we cannot determine the extent to which medical professionals follow the diagnosis recommendations of CDC and AAP. Further, the patient and hospital characteristics associated with SBS diagnosis codes remain unknown. Having this data could help researchers and policymakers identify the factors associated with SBS diagnosis. In response, we investigated seventeen-year trends in possible SBS, confirmed SBS, total SBS, and non-SBS abuse diagnosis codes among young children. We also examined the patient and hospital characteristics associated with these diagnosis codes.

SBS and AHT remain difficult social phenomena to surveil due to several factors, including the lack of mechanisms to detect mild cases in the general population, the reluctance of caregivers to come forward when AHT occurs, and discretion of professionals diagnosing SBS and AHT (including training, attitudes and controversy). Though diagnosis codes are an approximation of SBS, it is likely that the use of diagnosis codes is an imprecise measure of the actual incidence of SBS. Despite this limitation, these codes and setting are the best epidemiological data available for surveilling non-fatal SBS to our knowledge. In our study, we found support for the following: 1) Non-SBS abuse is the most common form of abuse in our study; 2) Confirmed SBS diagnosis trends have declined while possible SBS diagnosis trends have increased and total SBS trends remained stable; 3) All abuse diagnosis codes were more common among infant, male, or low-income children and urban teaching hospitals. Taken together, our findings contribute to literature on AHT and SBS along with non-SBS abuse diagnosis codes within hospitals, including diagnosis trends and characteristics associated with each abuse category. Policymakers can use our findings to develop plans for aligning current diagnostic practices with CDC and AAP guidelines.

According to our estimates, possible and confirmed SBS diagnosis codes represent a fraction of the overall abuse codes young children receive, with total SBS diagnosis codes comprising about 21% of abuse hospitalizations. Though diagnosis codes on possible and confirmed SBS in no way indicate the presence of abuse, diagnosis codes provide a rough estimate of SBS among young children hospitalized for abuse. A majority of hospitalizations were related to non-SBS abuse. Among children age 1 or younger, there was an overall increase in the probability of non-SBS abuse, whereas the probability of non-SBS abuse among children older than 1 year remained fairly stable. Hospitalizations often represent the most severe incidences of non-fatal child abuse, which are disproportionately experienced by young children (Farst et al. 2013). Similar to our results, researchers have reported stable overall trends in child maltreatment hospitalizations among children ages 0 to 3 from 1997 to 2009 (Wojciak et al. 2020). Farst and colleagues reported unchanging rates of child abuse hospitalizations among children ages 0 to 18, with young children facing the largest share of hospitalizations between 1998 and 2016 (Farst et al. 2013). Along with these study findings, our results provide evidence that child abuse hospitalizations remain a significant problem in the US, and may be a growing problem among young children.

Of our four abuse diagnosis categories, only confirmed SBS diagnosis codes declined between 2002 to 2014. This finding aligns with AHT literature indicating that AHT diagnosis codes decreased during overlapping time periods (2003–2008 (Parks et al. 2012); 2000–2009 (Shanahan et al. 2013)). There may be various explanations for reductions to confirmed SBS diagnosis codes over time, including declines in the actual incidence of SBS. Unfortunately, our findings on the decreasing use of SBS code 995.55 most likely does not reflect shifts in abusive caregiver practices. Given that possible SBS diagnose codes increased steadily over the study period and total SBS remained stable, a likelier explanation relates to changing diagnostic practices of medical professionals. Though AAP and CDC recommendations likely impacted the decline in confirmed SBS diagnosis codes, confirmed SBS diagnosis codes began decreasing in 2002, seven years before AAP’s recommendation in 2009. It is possible that medical and legal controversy surrounding the credibility of SBS diagnosis codes also contributed to declining use of SBS code 995.55. A few papers, for example, have questioned SBS as a reputable diagnosis, citing inadequate scientific evidence that the injuries typically associated with SBS are caused solely by shaking (Findley et al. 2019; Choudhary et al. 2019; Lyons 2003). The presence of these papers in medical research may have influenced physicians’ use of confirmed SBS diagnosis codes.

Whereas confirmed SBS diagnosis codes have decreased, possible SBS diagnosis codes have increased. By 2011, the rate of possible SBS diagnosis codes exceeded the rate of confirmed SBS diagnosis codes. This finding suggests that medical professionals are not diagnosing retinal hemorrhage and/or convulsions not associated with a seizure disorder, as confirmed SBS, even in the presence of physical abuse and Type 1 internal traumatic brain injury. If these diagnosis codes represent SBS yet are not coded as SBS, there are implications for diagnostic norms for AHT. At present, CDC-recommended AHT codes do not include retinal hemorrhage and/or convulsions without seizure disorder (i.e. possible SBS). The implication is that the diagnosis codes used to measure possible SBS may capture some cases, albeit a small amount, that would not meet the CDC’s definition of AHT (Center for Disease Control and Prevention 2012), perhaps leading to misclassification of AHT abuse, as previously indicated by Parks and colleagues (Parks et al. 2012).

Additionally, our findings suggest that the SBS diagnostic code 995.55 alone is not adequate to surveil for AHT, suggesting support for the use of the CDC’s AHT survelience codes. Although beyond the scope of this study, future researchers should investigate the overlap between AHT diagnoses codes and codes for retinal hemorrhages and convulsions without a seizure disorder, and compare trends in SBS diagnosis codes and AHT diagnosis codes over time. Future research should also examine whether current definitions of AHT neglect some diagnosis codes associated with abusive head injuries in young children (e.g., retinal hemorrhage and/or convulsions without seizure disorder in the presence of physical abuse and Type 1 internal traumatic brain injury). If current AHT definitions exclude some instances of abusive head injuries, policymakers could consider adding diagnosis codes to those recommended for AHT.

Our finding on increasing possible SBS diagnosis codes may also provide context to the overall process for diagnosing SBS, which Narang and Greeley describe as a complex, context-driven process without reputable diagnosis guidelines (Narang et al. 2020). Through our findings, there are a few directions researchers, policymakers, and medical professionals could take. If medical professionals find utility in diagnosing SBS, it appears that clearer diagnostic guidelines are needed, especially in light of the hospital characteristics associated with possible versus confirmed SBS diagnosis. According to our study, possible SBS diagnosis codes were more frequent in urban teaching hospitals and large hospitals, suggesting different diagnostic protocols in these institutions. Findley and colleagues recommend the development of a national registry on SBS and protocols for diagnosing SBS along with alternative explanations for SBS-like injuries (Findley et al. 2011). Likewise, we propose that researchers and pediatric medical providers agree to a standardized definition and diagnostic guidelines for possible and confirmed SBS, much like the AHT guidelines proposed by CDC, which may help reduce discrepancies in diagnosis and treatment and improve options for surveillance (Kim et al. 2017b; Paine et al. 2016).

Finally, our findings on the patient and hospital characteristics associated with SBS diagnosis codes align with what is known about AHT in the literature. Like AHT, our findings indicate that all abuse types were diagnosed more frequently among infants (< 1-year-old), boys, and children from low-income households than toddlers, girls, and children from higher-income households (Shanahan et al. 2013; Parks et al. 2012; Leventhal et al. 2012). The prevalence of SBS and AHT among infants under the age of 1 may be related to infant crying and subsequent parental or caregiver stress. Explanations for the prevalence of SBS among male infants may include the acoustic characteristics of male cries, societal norms related to crying in boys, gender stereotypes and biological differences. One recent study found that adult male caregivers were more aggravated by the cries of male infants than females (Richey et al. 2020). Future research may benefit from examining differences in SBS and AHT hospitalizations by child and perpetrator sex, given that the NIS data set includes codes for perpetrator’s gender and relationship to victim (e.g., abuse by father/step-father and abuse by mother/step-mother). Societal norms and gender stereotypes around crying may influence physicians and medical professionals diagnosis codes of abuse related injuries. For example, Ravichandiran and colleagues reported that physicians initially miss the abuse of boys more often than girls (Ravichandiran et al. 2009), suggesting that physicians may perceive injuries differently among boys and girls, possibly because boys are socialized for rough-and-tumble play and are more prone to accidental injury (Hagan and Kuebli 2007). Finally, biological differences between girls and boys may also account for the use of SBS diagnosis codes with boys: As compared to girls, boys are vulnerable to benign external hydrocephalus, predisposing children to subdural hemorrhages, and to subdural hemorrhages overall, which are attributed to SBS but can occur in the absence of shaking as well (Högberg et al. 2018; Wester 2019).

In all, we contribute to the literature by examining seventeen-year trends of SBS among young children hospitalized for abuse, yet there are limitations. First, our analysis includes no correction for confounders in the estimation of time trends. The lack of research on these trends, however, warranted our approach of estimating simple time trends by subgroup. Second, our possible SBS measure does not account for all SBS victims who are hospitalized nor SBS victims who remain undetected in the general population. Second, there are limitations associated with the sample and use of e-codes. One is that our sample does not include victims of SBS who were not hospitalized, including mild or fatal cases. Another is that SBS-related diagnostic codes are incomplete measures of SBS diagnosis, and do not include other factors of SBS diagnosis, including client history nor results from physical exams, ophthalmologic exams and radiological studies. As such, our results do not precisely represent the number of children experiencing SBS and instead provide information about the use of SBS diagnosis codes, an imprecise estimate of SBS diagnosis codes. Given the difficulty in surveilling SBS in the general population, we must rely on existing secondary data like the NIS to approximate the prevalence of SBS. Finally, SBS-related diagnosis codes only describe how medical professionals code abusive head injuries and cannot describe trends related to abusive parenting practices. Surveillance of child abuse itself remains a significant challenge in literature on child maltreatment, and is an issue our study cannot address (Fallon et al. 2010).

## Conclusion

Our study findings demonstrated that while confirmed SBS has decreased since 2002, possible SBS has increased. The discrepancy between trends in possible and confirmed SBS suggests differences in norms for diagnosing SBS, which has implications for which cases are considered AHT and which are not. Future research should investigate diagnostic processes for SBS and whether all codes associated with abusive head injuries in young children are being classified as AHT. Our findings also highlight the relativity defining and diagnosing SBS. According to our findings on confirmed SBS diagnosis codes, medical professionals find utility in the diagnosis, though may be more apt to apply possible SBS diagnosis codes to abusive head injuries in children given AAP and CDC recommendations and controversy surrounding SBS diagnosis codes. Clarifying norms for SBS diagnosis and refining definitions for AHT will ensure that all young children presenting with abusive head injuries included in overall counts of AHT. This baseline data, an essential component of child abuse surveillance, will enable ongoing efforts to track, prevent, and reduce child abuse.

## Supplementary Information

**Additional file 1: Appendix.** ICD-9-CIM Child Maltreatment Codes.

## Data Availability

The data that support the findings of this study are available for purchase from the Healthcare Utilization Project. Data are available, however, from the authors upon reasonable request and with permission of the Healthcare Utilization Project.

## Abbreviations

AAP: American Academy of Pediatrics
AHT: Abusive head trauma
CDC: Center for Disease Control and Prevention
CI: Confidence interval
ICD9-CM: International classification of diseases, clinical modification, ninth revision
NIS: National Inpatient Sample
SBS: Shaken baby syndrome
TBI: Traumatic brain injury
QIC: Quasi-likelihood information criterion
