# Supplementary material for: Trends in shaken baby syndrome diagnosis codes among young children hospitalized for abuse
Source: Inj Epidemiol. 2021 Jul 19;8:46. doi: 10.1186/s40621-021-00334-w (PMC8287751; doi:10.1186/s40621-021-00334-w)
Supplement: Supplementary file 1 — Additional file 1: Appendix. ICD-9-CIM Child Maltreatment Codes. [file 40621_2021_334_MOESM1_ESM.docx]

| Appendix. ICD-9-CIM Child Maltreatment Codes | |
| --- | --- |
| **Child maltreatment diagnosis (i.e., intent) codes** | |
| 995.50 | Child abuse unspecified |
| 995.51 | Child emotional or psychological abuse |
| 995.52 | Child neglect |
| 995.53 | Child sexual abuse |
| 995.54 | Child physical abuse |
| 995.55 | Shaken baby syndrome |
| 995.59 | Other child abuse or neglect |
| **External cause of injury (i.e., perpetrator) codes** | |
| E967.0 | Abuse by father or step-father |
| E967.1 | Abuse by other specified person |
| E967.2 | Abuse by mother or step-mother |
| E967.3 | Abuse by spouse or partner |
| E967.4 | Abuse by child |
| E967.5 | Abuse by sibling |
| E967.6 | Abuse by grandparent |
| E967.7 | Abuse by other relative |
| E967.8 | Abuse by non-related caregiver |
| E967.9 | Abuse by unspecified person |
| **Injury type diagnosis codes** | |
| 362.81 | Retinal hemorrhage |
| 780.39 | Convulsions not associated with a seizure disorder |
| 800-804 | Closed fracture of vault of skull (800), closed fracture of the base of skull (801), other closed and unqualified skull fractures (803), or closed fractures involving skull or face (804), with:   - cerebral laceration and contusion (.1) - subarachnoid subdural and extradural hemorrhage (.2) - unspecified intracranial hemorrhage (.3) - intracranial injury of other and unspecified nature (.4) - no mention of intracranial injury but with moderate loss of consciousness [1-24 hours] (.03) - no mention of intracranial injury but with prolonged loss of consciousness [more than 24 hours] (.04) - no mention of intracranial injury but with prolonged loss of consciousness [more than 24 hours], without return to pre-existing level of consciousness (.05)   Open fracture of vault of skull (800), open fracture of the base of skull (801), other open and unqualified skull fractures (803), or open fractures involving skull or face (804), with:   - cerebral laceration and contusion (.6) - subarachnoid subdural and extradural hemorrhage (.7) - unspecified intracranial hemorrhage (.8) - intracranial injury of other and unspecified nature (.9) - no mention of intracranial injury but with moderate loss of consciousness [1-24 hours] (.53) - no mention of intracranial injury but with prolonged loss of consciousness [more than 24 hours] (.54) - no mention of intracranial injury but with prolonged loss of consciousness [more than 24 hours], without return to pre-existing level of consciousness (.55) |
| 850 | Concussion with:   - moderate loss of consciousness (.2) - prolonged loss of consciousness (.3) - prolonged loss of consciousness without returning to pre-existing level of consciousness (.4) |
| 851-854 | Cerebral laceration and contusion (851), subarachnoid subdural and extradural hemorrhage following injury (852), other and unspecified intracranial hemorrhage following injury (853), or intracranial injury of other and unspecified nature (854) |
| 950 | Injury to the optic nerve pathways with:   - injury to the optic chiasm (.1) - injury to optic pathways (.2) - injury to visual cortex (.3) |
